# Factors Influencing Implementation of Family-Centered Care in a Neonatal Intensive Care Unit

**DOI:** 10.3389/fped.2020.00222

**Published:** 2020-05-06

**Authors:** Sabine M. Oude Maatman, Kajsa Bohlin, Siri Lilliesköld, Håvard T. Garberg, Irina Uitewaal-Poslawky, Marijke C. Kars, Agnes van den Hoogen

**Affiliations:** ^1^Department Woman and Baby, Wilhelmina Children's Hospital, University Medical Centre Utrecht, Utrecht, Netherlands; ^2^Department of Neonatology, Karolinska University Hospital, Stockholm, Sweden; ^3^Department of Pediatrics, Drammen Sykehus, Drammen, Norway; ^4^Clinical Health Science, Utrecht University, Utrecht, Netherlands

**Keywords:** family-centered care, FCC, neonatal intensive care unit, NICU, implementation

## Abstract

**Background:** Approximately 10% of all births worldwide are preterm. Often these infants are admitted at a Neonatal Intensive Care Unit (NICU). The NICU environment with periods of unnatural light, noise and repeated disturbances is very stressful for infants admitted to the NICU. In addition separation of parents causes stress for both infant and parents. A way to support and include parents in the care for their infants is Family-Centered Care (FCC). FCC is an approach of planning, delivery and evaluation of healthcare, based on a partnership between healthcare professionals and families of patients. Parents of infants who were admitted to an FCC unit were less stressed compared to parents at a Standard Care unit.

**Aim:** Although FCC is beneficial to families and patients, implementation can be challenging. Therefore it is important to know which factors can contribute or withhold the implementation of FCC. This study explored factors that influence implementation of FCC in NICU's according to healthcare professionals that work in a NICU with the concept FCC.

**Method:** A descriptive generic qualitative design with semi-structured interviews and inductive thematic analyses was used. This international multi-center study was conducted in three hospitals in three European countries: Sweden, Norway, and The Netherlands.

**Results:** Seven neonatal care nurses, one nurse assistant, five neonatologists, and three managers participated in this study. Four aspects were identified, when analyzing the data, namely: Behavioral change in staff, Family needs, Environment, and Communication. Most important is that almost all healthcare professionals described that the mind-set of the professional influences the implementation of FCC.

**Conclusion:** The mind-set of healthcare professionals in seeing parents as primary caregiver influences the way FCC is practiced and how parents are involved in the care for their infant.

## Highlights:

- Seeing parents as primary caregiver is an important mind-set of healthcare professionals when implementing FCC.
- The mind-set of healthcare professionals could be influenced by educating them about values of FCC.
- It is important, before implementing FCC successfully; management should be aware and invest in their staff.

## Introduction

Today's NICU environment reflects advances in technology and medical treatment of preterm and sick newborns. Approximately 7 infants per 100 births are admitted to a Neonatal Intensive Care Unit (NICU) (1). The NICU environment is stressful, with periods of unnatural light and noise, repeated disturbances caused by caretaking procedures and interference with parent-child interactions (2). In addition separation from parents may cause stress for both infant (3) and parent (4). A way to support and include parents in the care for their infants is Family-Centered Care (FCC). FCC is an approach in planning, delivering and evaluating healthcare, based on a partnership between healthcare professionals and families of patients (5). FCC distinguishes four basic values: dignity and respect, information sharing, family participation in care, and family collaboration (5). It aims that families should be included in the planning, implementation and evaluation of care and that their opinions should be as important as those of the health care professionals (6).

Research in neonatology shows a variety of beneficial effects of FCC for both infants as well as parents. It is beneficial in reducing length of hospital stay for infants admitted to a NICU (7), improves neurobehavioral outcomes in preterm infants (8), infants tend to grow faster (9), and infants are more likely to get breastfeeding at discharge (9). Moreover, parents of infants who were admitted to an FCC unit were less stressed than parents of infants admitted to a Standard Care unit (9). Also parents who participated during rounds, had easier access to information, were well-informed, more satisfied and could take part in the decision-making process (10).

Historically, NICUs were designed to support the medical care of infants and meet staff needs. There was little attention to the effects of the environment or care practices on infants and little effort was made to welcome and provide support to families (11). This typical NICU design does not support the relationship between infant and family (5). To be effective in FCC it is essential that the NICU is designed in such a way that families feel comfortable to spend prolonged periods of time with their infant and encouraged to engage in intimate, nurturing encounters (11).

Although FCC is beneficial for infants and families, implementation can be challenging, because the role of healthcare professional shifts from primary caregiver to being a mentor (12). Research shows that this leads to tension between parents and nurses, although nurses involve parents in care, they still want to retain control (12). Therefore communication is of importance for good partnership between parents and healthcare professionals. Whereas insufficient communication may lead to role stress, negotiation failure, feelings of insecurity and stress by both, parents and nurses (13, 14).

For a successful implementation of FCC in the NICU, it is important to explore factors that influence implementation of FCC. Implementation is defined as the introduction of an innovation in to daily routine. This demands effective communication strategies and removal of barriers to change, by using effective educative and policy techniques (15). FCC is successfully implemented when care is delivered based on the principals of FCC and families and staff are satisfied working with it. Knowing which factors may contribute or withhold a successful implementation makes it easier to decide which strategies should be taken to implement this concept successfully in a neonatal setting. Therefore the aim of the study is to explore factors influencing implementation of Family-Centered Care NICU's among three different northern European countries.

## Methods

### Design

A descriptive qualitative design with semi-structured interviews using a thematic analysis (16).

### Population and Setting

The populations of interest are healthcare professionals and managers working on a NICU that adopted FCC. Healthcare professionals were eligible to participate when they (I) worked as healthcare professional or manager in a NICU, (II) speak fluent in English or Dutch, (III) had at least one year of working experience with FCC in their current profession, and (IV) worked at least 16 h a week.

This study was conducted in three level III NICU's in different hospitals, located in urban areas in Sweden, Norway and The Netherlands. All three hospitals deliver post intensive care in addition. Karolinska university hospital, Sweden provides care for infants born from 22 weeks of gestation. Vestre Viken, a general hospital in Norway, provides care for infants born from 28 weeks of gestation. Maxima Medical Centre, a general hospital in The Netherlands, provides care for infants born from 24 weeks of gestation.

All included hospitals implemented FCC and subsequently rebuild their wards between 2010 and 2012; including rooming-in or sleeping facilities for parents near their infants.

### Sampling

Allowing to maximize the collection of available data, insights, and factors that influence the implementation of FCC, a purposeful sample consisting of healthcare professionals working at one of the NICU's included in this study, was used (17). Maximum variation was sought with respect to professional role and work experience in FCC. Therefore, neonatal care nurses, nurse assistants, neonatologists, and managers were included.

Recruitment started in February 2018. Selection of eligible participants was done by a contact person in each hospital at site and the responsible researcher. Eligible participants equally distributed per hospital were approached by the researcher through email. This email contained brief information about the study, contact details of the researcher and an invitation to participate in the study. When a participant was interested to participate, the information letter was sent and the interview was scheduled.

### Data Collection

Data were collected in February and March of 2018 using semi-structured, face-to-face interviews. Before the start of data collection, three pilot interviews were conducted, to enable refinement of the interview guide, and to improve skills of the responsible researcher who performed the interviews.

An interview guide was constructed based on literature regarding perspectives and expectations of FCC (12, 18–21). The interview started with a short introduction followed by the opening question: *How did you experience the implementation of FCC at your ward?* Main subjects were: involvement of family in the care, caring for the family, legislation, and resources. Baseline characteristics were obtained before the interview. The participants were interviewed in the hospital of employment, in a private room on the ward. All interviews were digitally recorded. All interviews were conducted by the responsible, first researcher.

### Data Analysis

Baseline characteristics were analyzed using IBM SPSS Statistics (IBM, North Castle, New York, United States). All the interview data was managed in NVivo (QSR International Pty Ltd. NVivo Qualitative Data-analysis Software. Melbourne, Australia). A thematic analysis following a six phase model of Braun and Clark (16) was used to identify influencing factors on the implementation FCC.

In the first phase, “familiarizing yourself with your data,” the data from interviews were transcribed verbatim. Transcribing the data, reviewing the data line by line and reading through the entire data set helped the researcher become familiar with the data. In the next phase, “generating initial codes,” the researcher generated codes for all potential categories or patterns.

Data collection and analysis is an iterative process. Constant comparison is used to determine commonalities and variations and adjustment of the interview guide. Meaning that codes and themes elicited from the “new” data will be constantly compared to previously collected data (17, 22). Coding and generating initial themes is done by two researchers, differences were discussed until consensus was reached about codes and initial themes.

In the following phases, “searching for themes,” “reviewing themes,” and “defining and naming themes,” the list of codes were analyzed to determine how each code may fit into an overarching theme. Each theme was identified and analyzed in relation to the aim of the study. Phase six, “producing the report,” followed after completing the aforementioned phases.

All transcripts were sent for member check to participants.

### Ethical Issues

The study was conducted according to the World Medical Association Declaration of Helsinki (23). The Medical Research Ethics Committee has stated that this study did not fall within the scope of the Dutch Medical Research Involving Human Subjects Act (WMO). Protocol ID: 18-083.

## Results

### Sample

Of 20 invited healthcare professionals, sixteen agreed to participate in the study. Amongst them were neonatal care nurses (*N* = 7), nurse assistant (*N* = 1), neonatologists (*N* = 5), and managers (*N* = 3). See Table 1 for baseline characteristics. The duration of the interview varied from 25 min to 1 h.

**Table 1:** Baseline characteristics Healthcare professionals (*N* = 16).

	***N***	**%**	**Mean (SD)**
**Age**			45.8 (6.28)
**Gender**			
Male	0	0	
Female	16	100	
**Profession**			
Nurse	7	43.8	
Assistant Nurse	1	6.3	
Neonatologist	5	31.3	
Manager	3	18.8	
**Employed**			
Part-time	4	25	
Full-time	12	75	
**Hospital**			
Sweden	6	37.5	
Norway	5	31.25	
The Netherlands	5	31.25	
**Work Experience (years)**			20.44 (7.17)
**Work Experience FCC (years)**			7.56 (3.76)

### Main Results

The following four themes were identified: (1) Behavioral change in staff, (2) Family needs, (3) Environment, and (4) Communication. Some themes are based on several different categories.

### Behavioral Change in Staff

#### Mind-Set

All healthcare professionals described that FCC required a mind-set off working. It's a mind-set to see the parents as primary caregiver for the infant and to involve them in the care. Having this mind-set is important in practicing FCC, because it enhances the involvement of parents in the care. Healthcare professionals described that they already had this mind-set before FCC was implemented. For them the implementation of FCC was a natural step in the care for the infant and their family. “*Parents are the primary care giver, they are the ones that take care of the babies. of course, of course of course!” (SWNU05)* Being a mentor for parents instead of primary caregiver is considered a big change in daily care and is not how healthcare professionals traditionally are educated. “*.intellectually we would all agree that parents are the ones that should and ought to be, and are closest to their infants […] but traditionally that's not the way we have organized our care.” (SWMA07)* Healthcare professionals described that preparing and guiding them for this change is crucial.

#### Creating the Mind-Set

All managers described that they tried to influence the mind-set of the healthcare professionals by educating them in FCC and sharing scientific evidence with them, to achieve a better understanding of the importance of FCC. Healthcare professionals also described that having reflection sessions with their colleagues where they could express their ideas and concerns about FCC helped them to get a better understanding of FCC. Furthermore, all managers and most of the healthcare professionals described that it is important to involve the staff in the implementation of FCC, from the first idea, until the actual implementation.

“‘But it has all to do with in informing them (staff), sharing with them and involve them in the development.”(NLNU13)

Healthcare professionals expressed that having examples from other hospitals that have experience with FCC, would have helped them to better understand what is expected from them in their new role within FCC. Also to see that it is possible to deliver care according to the FCC principals. “*I think, the first thing is that they (healthcare professionals) know that it's possible.” (NODR12)*

#### Motivation

Most of the healthcare professionals described that seeing what FCC meant for infants and parents, had motivated them during the implementation of FCC. Positive experiences from parents with FCC, shared with healthcare professionals, stimulated healthcare professionals to continue with FCC. They described that it made them aware of how important it was for infant and family. “*But when you see the parents, how the outcome is for them. You never want to do anything else, no really!”(NONU10)*

Another factor that stimulated the staff in all three hospitals was the publicity and attention they had after implementing FCC. Healthcare professionals from other hospitals and other countries came over to visit their ward, to see how FCC was practiced and they became a role model for other hospitals.

### Family Needs

#### Participation in Care

Important to conduct FCC, is involvement of parents in the care for their infant's. All interviewed healthcare professionals involve parents in caring for their infant. Some doctors, nurses and managers state that it helps them to involve parents in the care if doctors have a conversation with parents, before the infant is born. “*…we meet them (parents) before they give birth to preterm babies. We inform them already at that stage of how the FCC would be carried out. Why we believe it is important.” (SWDR06)* This conversation contains information about what parents can expect of their infant after birth, what parents can do to support their infant and how important it is that parents are involved in the care.

#### Medical Rounds

Among participants there was no consensus in involving parents in medical rounds. Sweden has medical rounds where parents are included. In Norway and The Netherlands, parents are not included in the medical rounds. Some of the doctors in Norway and The Netherlands described that it might be a burden for parents to hear about possible diagnoses and complications. “*Because I don't think it's necessarily thing to drag every diagnose close up to the parents and then later evaluate it. I think it's not a very psychologically thing to do.”(NODR11)*

Doctors in Sweden and most nurses and managers are positive about the involvement of parents during rounds. They think it is natural to include them, because parents know their infant best. “*They are more informed on what is going on, we have no secrets, the feel secure of that, we don't have secrets. And we also pick their questions or their worries up very quickly.”(SWDR06)*

### Environment

#### Facilities on the Ward

Healthcare professionals described that the ward is of influence in practicing FCC. It gave them the final step to optimize FCC. Healthcare professionals described that it is important for parents to have a place on the ward where they can stay and sleep, preferably close to their infant. Professionals mentioned that having single rooms and a private bathroom is important. The ward should provide everything that parents need on a daily basis, like a canteen or kitchen, a washing machine and a place where they can withdraw, away from the infant. “*Because for everything what you remove or not take into the unit, the parents have to leave the unit.”(NONU09)*

Most of the professionals mentioned that the ward must give the parents a welcoming feeling and the environment should not look like a hospital.

#### Legislation

Healthcare professionals in Sweden and Norway described that parents are continuously present. This helps them to involve parents in the care. Legislation in these countries makes it possible for parents to stay in the hospital with their infant. In The Netherlands however, fathers have to go back to work one week after the infant is born. Healthcare professionals in The Netherlands described that it is not common that parents are rooming-in. “*But then I wonder, in some countries they manage that parents are with their infant, up to 8 hours a day! And why can't we make that happen here?”(NLDR15)*

#### Communication

Healthcare professionals described that communication is important in FCC. Communication with parents about the care of their infant, communication in guiding, and coaching parents in the care for their infant and communication in supporting and facilitating parents in their needs. Most of the professionals described the communication with parents as an “open communication.”

However, most nurses experienced changes in communication with parents, because parents are almost continuously present in FCC. Therefor nurses are more confronted with parents who are in a crisis because their infant is born too early. For that reason, nurses have to deal with different emotions from parents, like anger and sadness. Nurses described that they needed some extra tools in how to deal with parents in crisis. “*To communicate good with parents in crisis is a bit hard.”(NONU10)*

## Discussion

This study shows that the main important finding influencing the implementation of FCC, was behavioral change in staff, to see parents as primary caregiver. A different mind-set of the healthcare professional needs to be created. This study also found that factors influencing the implementation of FCC are: Behavioral change in staff, Family needs, Environment, and Communication.

The study highlights the importance of preparing the staff in the transition from primary caregiver to a mentor for parents. Healthcare professionals are traditionally not educated to deliver care according to FCC principals. Trajkovski et al. (12) concluded that education on its own is not enough. Healthcare professionals need ongoing organizational support and guidance to deliver FCC in the NICU (12). Within the theme “communication” healthcare professionals in this study described that having parents constantly present makes it sometimes difficult to communicate with parents. Guiding and coaching parents is more than simply giving information about the technical and medical aspects. A recent quantitative study among healthcare professionals' view on parent participation in the NICU (24) supports this finding by concluding that it is important that nurses as well as doctors need training in communication skills to more effectively support and encourage parental participation (24). This shows that it is important to train healthcare professionals in communication so they have some extra tools to communicate well with parents and family.

The theme “family needs” in this study described that nurses adapt their level of support based on a conversation they have with parents. They feel that this is sufficient in supporting parents in taking care for their infant. However, research that studied the perceptions of parent support by parents, nurses, and physicians revealed that it is important that healthcare professionals are encouraged to critically reflect on whether the type and consistency of support they provide to parents is in line with parents' perceptions and needs (25). Moreover, other studies suggest that families experience emotional support as inadequate and value staff that empathizes with their situation (18, 26). Maybe this finding can be explained by the fact that nurses may think that they are providing support, but families may not experiences this as support.

Another factor discussed in the theme “family needs” is whether parents must be included in the medical rounds or not. In this study no consensus was found concerning the participation of parents in the medical rounds. Especially doctors in this study expressed concerns about this involvement, because of the burden for parents of hearing possible diagnoses and complications. However, currently available evidence suggests that parents are less concerned with the stress imposed by rounds compared to their need for information. When given the choice, between 85 and 100% of the parents would prefer to be present at rounds (19). Healthcare professionals should let parents decide if they want to be involved in the medical rounds, instead of deciding for them.

Within the theme “environment” the design of the ward was discussed. Healthcare professionals state that the design of the ward gave them the final step in optimizing FCC. Research about ward design reveals that a single patient room is perceived by parents as an improvement in privacy and the design can complement an implemented concept of FCC (27). Moreover, parents in single patient rooms where more involved in care (28). However, these results are related to single room versus an open bay unit. To the author's knowledge, there is currently no available research about the facilities that are offered on a ward, like a kitchen or laundry facilities for parents that might support FCC.

### Strengths and Limitations

There are some limitations in this study. First, due to the variety of the sample, including several disciplines of healthcare professionals form three hospitals, data saturation was not achieved on all themes, except in “behavioral change of staff.” Therefore, the results must be considered with caution. To increase transferability, future studies should include more healthcare professionals. Second, selection bias might have occurred, because all participating healthcare professionals were selected by the contact person of the included hospitals. The participating healthcare professionals might have been selected because of their positive attitude towards FCC and eagerness to participate in this study. Third, the time between the implementation of FCC and the interview can cause recall bias. In some cases the time between the implementation of FCC and the interview was ten years. Therefore participants might think more positive about the implementation based on their current experiences with FCC. Fourth, the researcher works as a nurse at a NICU, this might have influenced the objectivity of the data collection (29). However, an interview guide is used aiming to improve the quality and objectivity of the data collection (29).

A strength of the study is that the trustworthiness (29) was improved by two researchers that independently coded all the interviews and created initial themes. Together they reached consensus about the final themes. Other methods to improve trustworthiness were: recording interviews, a member check of the interview transcripts, peer review and transparent reporting following the COREQ guidelines (30).

### Conclusion and Recommendations

The mind-set of healthcare professionals in seeing parents as primary caregiver is important. This influences the way FCC is practiced and how parents are involved in the care. A recommendation for clinical practice is to influence this mind-set by showing healthcare professionals why FCC is important. This could be done by providing a literature overview with scientific evidence about outcomes for infants and families. In addition, short internships in hospitals that are experienced in FCC should be introduced, to experience what FCC means for infants, families and healthcare professionals. Further research regarding ward design should also consider facilities that are offered on the ward that might support families.

## Data Availability Statement

The datasets generated for this study are available on request to the corresponding author.

## Ethics Statement

The studies involving human participants were reviewed and approved by The Medical Research Ethics Committee University Hospital Medical Centre Utrecht. The participants provided their written informed consent to participate in this study. Written informed consent was obtained from the individual(s) for the publication of any potentially identifiable images or data included in this article.

## Author Contributions

All persons designated as authors are qualified for authorship, and are listed below. Each author has participated sufficiently in the work to take public responsibility for appropriate portions of the content. SO: conceptualization, data collection, analysis, investigation, methodology, project administration, software, validation, visualization, writing, review and editing. KB and SL: investigation, resources, supervision, review and editing. HG: supervision, resources, review and editing. IU-P and MK: conceptualization, review and editing. AH: conceptualization, analysis, investigation, methodology, resources, software, supervision, validation, writing, review and editing.

## Conflict of Interest

The authors declare that the research was conducted in the absence of any commercial or financial relationships that could be construed as a potential conflict of interest.

## Abbreviations

FCC: Family-Centered Care
NICU: Neonatal Intensive Care Unit.

## References

[1] Harrison WN Wasserman JR Goodman DC. Regional variation in neonatal intensive care admissions and the relationship to bed supply. J Pediatr. (2018) 192:73–79.e4. 10.1016/j.jpeds.2017.08.028 28969888

[2] Altimier L Phillips RM The neonatal integrative developmental care model: seven neuroprotective core measures for family-Centered developmental care. Newborn Infant Nurs Rev. (2013) 13:9–22. 10.1053/j.nainr.2012.12.002

[3] Bialoskurski M Cox CL Hayes JA. The nature of attachment in a neonatal intensive care unit. J Perinat Neonatal Nurs. (1999) 13:66–77. 10.1097/00005237-199906000-00007 10633666

[4] Cleveland LM. Parenting in the neonatal intensive care unit. J Obstet Gynecol Neonatal Nurs. (2008) 37:666–91. 10.1111/j.1552-6909.2008.00288.x 19012717

[5] Griffin T. Family-centered care in the nICU. J Perinat Neonatal Nurs. (2006) 20:98–102. 10.1097/00005237-200601000-00029 16508475

[6] Neal A Frost M Kuhn J Green A Gance-Cleveland B Kersten R. Family centered care within an infant-toddler unit. Pediatr Nurs. (2007) 33:481–5. 18196711

[7] Ortenstrand A Westrup B Broström EB Sarman I Akerström S Brune T. The stockholm neonatal family centered care study: effects on length of stay and infant morbidity. Pediatrics. (2010) 125:e278–85. 20100748 10.1542/peds.2009-1511

[8] Yu Y-T Hsieh W-S Hsu C-H Lin Y-J Lin C-H Hsieh S. Family-centered care improved neonatal medical and neurobehavioral outcomes in preterm infants: randomized controlled trial. Phys Ther. (2017) 97:1158–68. 10.1093/ptj/pzx089 29186633

[9] O'Brien K Bracht M Macdonell K McBride T Robson K O'Leary L. A pilot cohort analytic study of family integrated care in a canadian neonatal intensive care unit. BMC Preg Childbirth. (2013) 13(Suppl 1):S12. 10.1186/1471-2393-13-S1-S12 23445639 PMC3561192

[10] Aronson PL Yau J Helfaer MA Morrison W. Impact of family presence during pediatric intensive care unit rounds on the family and medical team. Pediatrics. (2009) 124:1119–25. 10.1542/peds.2009-0369 19736262

[11] Dunn MS MacMillan-York E Robson K Single family rooms for the nICU: pros, cons and the way forward. Newborn Infant Nurs Rev. (2016) 16:218–21. 10.1053/j.nainr.2016.09.011

[12] Trajkovski S Schmied V Vickers M Jackson D. Neonatal nurses' perspectives of family-centred care: a qualitative study. J Clin Nurs. (2012) 21:2477–87. 10.1111/j.1365-2702.2012.04138.x 22889445

[13] Coyne I. Families and health-care professionals' perspectives and expectations of family-centred care: hidden expectations and unclear roles. Health Expect. (2015) 18:796–808. 10.1111/hex.12104 23800327 PMC5060842

[14] Foster M Whitehead L Maybee P. Parents' and health professionals' perceptions of family centred care for children in hospital, in developed and developing countries: a review of the literature. Int J Nurs Stud. (2010) 47:1184–93. 10.1016/j.ijnurstu.2010.05.005 20646708

[15] Grol R Wensing M Eccles M Davis D Improving patient care the implementation of change in health care. John Wiley & Sons;. (2013) A 10.1002/9781118525975

[16] Braun V Clarke V Using thematic analysis in psychology. Qual Res Psychol. (2006) 3:77–101. 10.1191/1478088706qp063oa

[17] Boeije H Analysis in Qualitative Research. London, UK: SAGE (2010).

[18] Gooding JS Cooper LG Blaine AI Franck LS Howse JL Berns SD. Family support and family-Centered care in the neonatal intensive care unit: origins, advances, impact. Semin Perinatol. (2011) 35:20–8. 10.1053/j.semperi.2010.10.004 21255703

[19] Davidson JE. Family presence on rounds in neonatal, pediatric, and adult intensive care units. Ann Am Thorac Soc. (2013) 10:152–6. 10.1513/AnnalsATS.201301-006PS 23607848

[20] Beck SA Weis J Greisen G Andersen M Zoffmann V Room for family-centered care - a qualitative evaluation of a neonatal intensive care unit remodeling project. J Neonatal Nurs. (2009) 15:88–99. 10.1016/j.jnn.2009.01.006

[21] Boztepe H Kerimoglu Yildiz G Nurses perceptions of barriers to implementing family-centered care in a pediatric setting: a qualitativestudy. J Spec Pediatr Nurs. (2017) 22:e12175 10.1111/jspn.12175 28198079

[22] Creswell JW Creswell JW Qualitative Inquiry & Research Design : Choosing Among Five Approaches. Los Angeles, CA: SAGE Publications (2013).

[23] Kong H West S WMA declaration of helsinki - ethical principles for scienti c requirements and research protocols. World Med Assoc. (2013) 1964:29–32.

[24] Kjellsdotter A Lantz B Ottosson C Healthcare professionals' views on parental participation in the neonatal intensive care units. J Pediatr Nurs. (2017) 41:3–8. 10.1016/j.pedn.2017.09.008 28965802

[25] Franck LS Axelin A. Differences in parents', nurses' and physicians' views of nICU parent support. Acta Paediatr. (2013) 102:590–6. 10.1111/apa.12227 23463946

[26] Sarajarvi A Haapamaki ML Paavilainen E. Emotional and informational support for families during their child's illness. Int Nurs Rev. (2006) 53:205–10. 10.1111/j.1466-7657.2006.00479.x 16879183

[27] Bodack E Schenk O Karutz H Die einrichtung von einzelzimmern auf neonatologischen intensivstationen - auswirkungen auf die betreuung aus sicht der eltern. Z Geburtshilfe Neonatol. (2016) 220:124–9. 10.1055/s-0042-102886 27124738

[28] Shahheidari M Homer C. Impact of the design of neonatal intensive care units on neonates, staff, and families. J Perinat Neonatal Nurs. (2012) 26:260–6. 10.1097/JPN.0b013e318261ca1d 22843008

[29] Holloway I Wheeler S Holloway I Qualitative Research in Nursing and Healthcare. Fortaleza: Wiley-Blackwell (2010).

[30] Tong A Sainsbury P Craig J. Consolidated criteria for reporting qualitative research (COREQ): a 32-item checklist for interviews and focus groups. Int J Qual Heal Care. (2007) 19:349–57. 10.1093/intqhc/mzm042 17872937

